# Results after simple decompression of the ulnar nerve in cubital tunnel syndrome

**DOI:** 10.3205/iprs000078

**Published:** 2015-12-21

**Authors:** Kristina Harder, Sandra Lukschu, Sebastian E. Dunda, Björn Dirk Krapohl

**Affiliations:** 1 Department of Plastic and Hand Surgery, St. Marien-Krankenhaus Berlin, Germany; 2 Center for Musculoskeletal Surgery, Charité – Medical University of Berlin, Germany

**Keywords:** cubital tunnel syndrome, nerve decompression, DASH score

## Abstract

Cubital tunnel syndrome represents the second most common compression neuropathy of the upper limb. For more than four decades there has been a controversy about the best surgical treatment modality for cubital tunnel syndrome.

In this study the results of 28 patients with simple ulnar nerve decompression are presented. Data analyses refers to clinical examination, personal interview, DASH-questionnaire, and electrophysiological measurements, which were assessed pre- and postoperatively.

28 patients (15 females, 13 males) were included in this study. The average age at time of surgery was 47.78 years (31.68–73.10 years). The period from onset of symptoms to surgery ranged from 2 to 24 months (mean 6 months). The mean follow-up was 2.11 years (0.91–4.16 years).

Postoperatively there was a significant decrease in DASH score from 52.6 points to 13.3 points (p<0.001). Also the electrophysiological findings improved significantly: motor nerve conduction velocity increased from 36.0 m/s to 44.4 m/s (p=0.008) and the motor nerve action potential reached 5,470 mV compared to 3,665 mV preoperatively (p=0.018). A significant increase of grip strength from 59% (in comparison to the healthy hand) to 80% was observed (p=0.002). Pain was indicated by means of a visual analog scale from 0 to 100. Preoperatively the median level of pain was 29 and postoperatively it was 0 (p=0.001). The decrease of the two-point-discrimination of the three ulnar finger nerves was also highly significant (p<0.001) from 11.3 mm to 5.0 mm. Significant postoperative improvement was also observed in the clinical examination concerning muscle atrophy (p=0.002), clawing (p=0.008), paresthesia (p=0.004), the sign of Froment (p=0.004), the sign of Hoffmann-Tinel (p=0.021), and clumsiness (p=0.002).

Overall nearly 90% of all patients were satisfied with the result of the operation. In 96.4% of all cases, surgery improved the symptoms and in one patient (3.6%) the success was noted as “poor” because the symptoms remained unchanged. In 35.7% the success was graded as “moderate”, in 10.7% as “good” and in 50.0% as “very good”.

## Zusammenfassung

Das Sulcus-nervi-ulnaris-Syndrom stellt die zweithäufigste Kompressionsneuropathie der oberen Extremität dar. Seit über vier Jahrzehnten werden die verschiedenen Methoden der operativen Therapie des Sulcus-nervi-ulnaris-Syndroms kontrovers diskutiert.

In dieser Arbeit werden die Ergebnisse nach einfacher Dekompression bei 28 Patienten dargestellt. Anhand von klinischen Untersuchungen, persönlicher Befragung des Patienten, DASH-Fragebögen und elektrophysiologischen Befunden, die prä- und postoperativ erhoben wurden, wurden die Ergebnisse der Operation analysiert.

Das Patientenkollektiv bestand aus insgesamt 28 Patienten (15 weiblich, 13 männlich). Das mittlere Alter zum Operationszeitpunkt betrug 47,78 Jahre (31,68–73,10 Jahre). Zu diesem Zeitpunkt bestanden die Beschwerden seit durchschnittlich 6 Monaten (2–24 Monate). Der mittlere Nachbeobachtungszeitraum betrug 2,11 Jahre (0,91–4,16 Jahre).

Postoperativ kam es zu einer signifikanten (p=0,001) Abnahme des DASH Scores von 52,6 auf 13,3 Punkte. Auch die elektrophysiologischen Befunde besserten sich signifikant: die motorische Nervenleitgeschwindigkeit stieg von 36,0 m/s auf 44,4 m/s an (p=0,008) und die motorische Summenpotentialamplitude erreichte nach der Operation 5.470 mV im Vergleich zu 3.665 mV präoperativ (p=0,018). Es kam zu einem signifikanten Kraftanstieg im Grobgriff von präoperativ 59% (der Kraft der gesunden Hand) auf postoperativ 80% (p=0,002). Die Schmerzen vor der Operation lagen auf der visuellen Analogskala von 0 bis 100 bei einem Median von 29; postoperativ lag der Median bei 0 (p<0,001). Ebenfalls hoch signifikant war die Abnahme der Zwei-Punkte-Diskrimination der drei ulnaren Fingernerven von durchschnittlich 11,3 mm auf 5,0 mm (p<0,001).

Signifikante Verbesserungen konnten auch in der klinischen Untersuchung hinsichtlich der Atrophie (p=0,002), der Krallenstellung (p=0,008), den Parästhesien (p=0,004), dem Froment-Zeichen (p=0,004), dem Hoffmann-Tinel-Zeichen (p=0,021) und der Ungeschicklichkeit (p=0,002) gefunden werden.

Insgesamt waren knapp 90% der Patienten mit dem Ergebnis der Operation zufrieden. Bei 96,4% der Patienten wurde durch die Operation eine Verbesserung der Symptomatik erreicht und bei einem Patienten (3,6%) blieb der Zustand unverändert und wurde daher der Erfolg als „schlecht“ eingestuft. Bei 35,7% war der Operationserfolg „mäßig“, bei 10,7% „gut“ und bei 50,0% „sehr gut“.

Schlussfolgernd ist festzustellen, dass die einfache Dekompression eine bewährte, minimal invasive und effektive Methode zur Behandlung des Sulcus-nervi-ulnaris-Syndroms ist.

## Introduction

The cubital tunnel syndrome (CTS) is a frequent diagnosis for neuropathy of the upper limb. It is characterized by pain at the ulnar part of the forearm, paresthesia, and hypesthesia, accompanied by sensitive and motoric dysfunction. This leads to a severe disability and restrictions in daily life.

There are multiple treatment options concerning surgical interventions but still without an academic level of evidence for one of them. 

The investigators of this study present a long-term follow-up with a complex clinical and physiological testing scale after single decompression of the ulnar nerve in cubital tunnel syndrome.

## Methods and material

During a three year period 56 single decompression operations caused by cubital tunnel syndrome were performed in the institution. 28 patients could be randomized into the study. The mean follow-up was 2.1 years after operation and was realized by an extensive protocol of examinations. The patients were evaluated by a questionnaire, including the DASH score, visual analogue scale, and a general assessment on the subjective benefit of the operation and its effect. 

Furthermore, we examined the patients focusing on the parameters: grip strength (Jamar dynamometer), range of motion, functional thumb adduction (Froment sign), static two point discrimination, nerve irritation (Hoffmann-Tinel sign), and electroneurophysiological parameters.

## Results

There was an equal distribution of sex (53.6% female, 46.4% male). The mean age was 48 years and the persistence of symptoms until operation was 6 months (2–24). Remarkable was the affliction of the non-dominant hand:

The DASH score (Disability of the arm, shoulder and hand) was significantly reduced post-operatively (52.6 vs. 13.3; p<0.001) as well as the pain-scale and the feeling of clumsiness of the hand. Preoperatively the median level of pain was 29 and postoperatively it was 0 (p=0.001).

**Figure 1:**
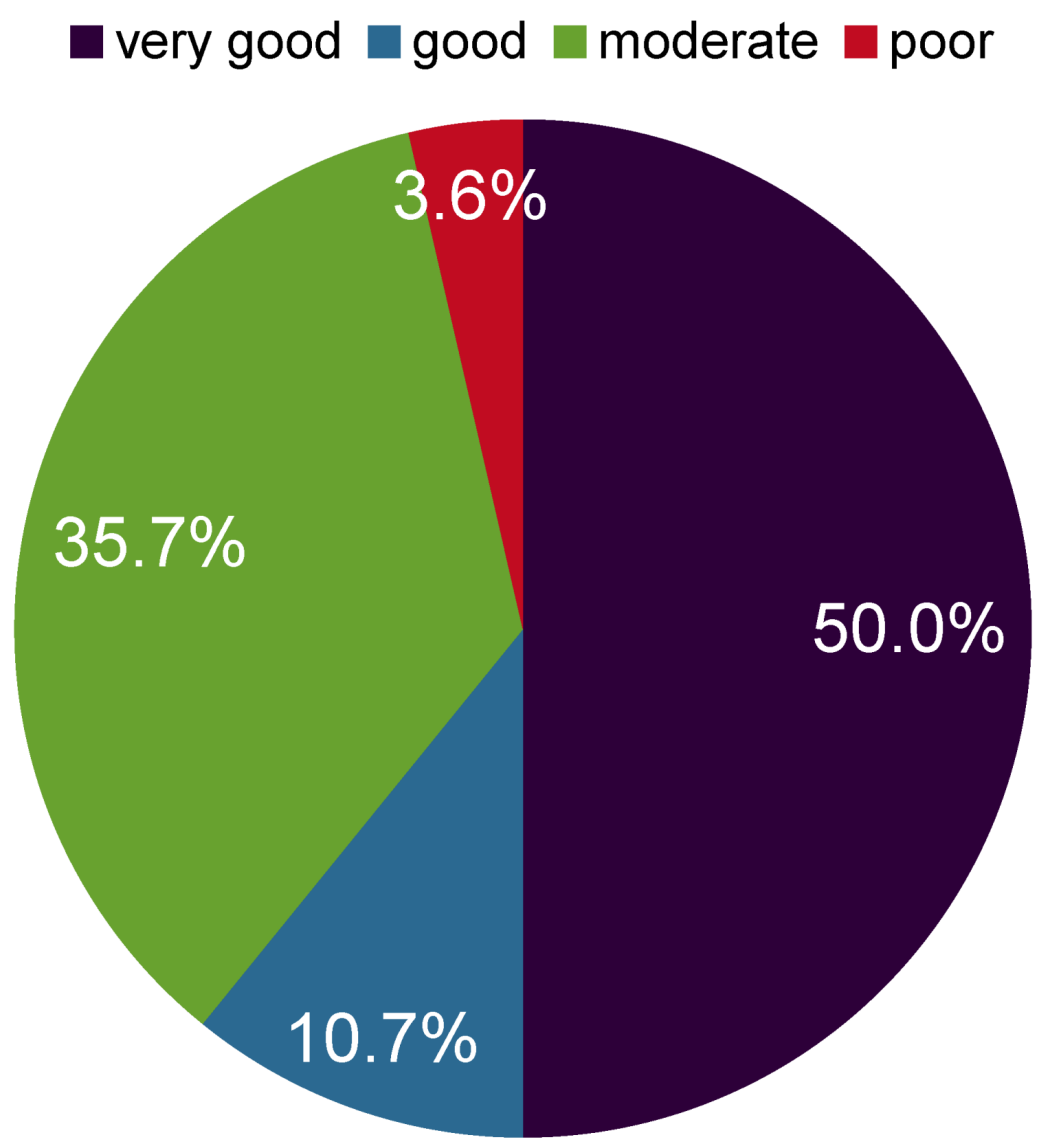
Patients’ rating of the result after nerve decompression in cubital tunnel syndrome

Concerning the technical examination grip strength measured by Jamar dynamometer showed an improvement. A significant increase of grip strength from 59% (in comparison to the healthy hand) to 80% was observed (p=0.002). Pain was indicated by means of a visual analog scale from 0 to 100. Preoperatively the median level of pain was 29 and postoperatively it was 0 (p=0.000). The decrease of the two-point-discrimination of the three ulnar finger nerves was also highly significant (p<0.001) from 11.3 mm to 5.0 mm. Significant postoperative improvement was also observed in the clinical examination concerning muscle atrophy (p=0.002), clawing (p=0.008), paresthesia (p=0.004), the sign of Froment (p=0.004), the sign of Hoffmann-Tinel (p=0.021), and clumsiness (p=0.002). Overall nearly 90% of all patients were satisfied with the result of the operation. In 96.4% of all cases, surgery improved the symptoms and in one patient (3.6%) the success was noted as “poor” because the symptoms remained unchanged. In 35.7% the success was graded as “moderate”, in 10.7% as “good” and in 50.0% as “very good” (Figure 1 (Fig. 1)).

Paraesthesia is one of the major symptoms and were found in 27 from 28 patients before the operation. This was also reduced significantly (p=0.004) by the operation, but 18 patients reported about any kind of paraesthetic feelings. 

Also the positive Hofmann-Tinel sign was persisting in 12 of 21 cases.

**Figure 2:**
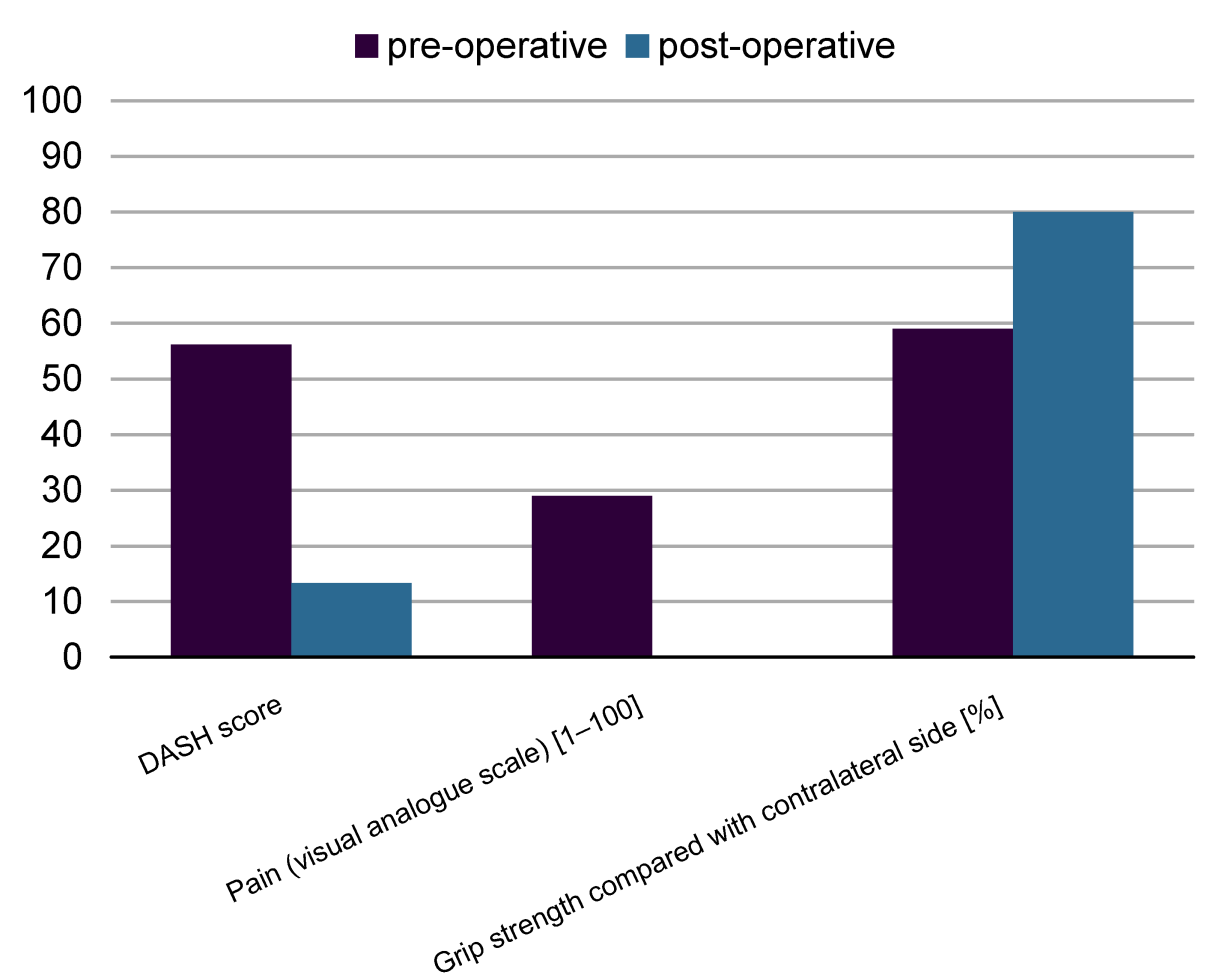
Summary of the most important parameters, comparing the state before and after ulnar nerve decompression in cubital tunnel syndrome

The most important parameters, DASH score, pain scale, and grip strength are summarized in Figure 2 (Fig. 2).

## Discussion

Even in the 21^st^ century, there are multiple uncertainties in diagnostic and treatment methods of cubital tunnel syndrome. Up to 30% of patients suffering from cubital tunnel syndrome are idiopathic and then often correlated with an additional carpal tunnel syndrome. The diagnostics even in case of neurophysiological testing do not necessarily correlate with the clinical findings and symptoms [1]. The benefit of the operation is reduction of pain, increase of motor und sensory function of the inflicted arm. The standard methods for surgery include simple nerve-decompression, different types of transposition of the ulnar nerve, and medial epicondylectomy. However, there is no evidence for any of them to deliver significantly better results than one of the other techniques [2], [3], [4].

There are multiple investigations concerning the treatment of the cubital tunnel syndrome but only rare studies are randomized or prospectively planned [5]. 

The single nerve decompression, open or endoscopically, is the operation with the lowest tissue trauma and the fewest damage to the Vasa nervorum [6], [7]. The transposition of the nerve is more complex and gets along with a higher incidence of peri-operative complications [3].

This study confirms the excellent outcome after single decompression of the ulnar nerve. The investigators propose this method as „gold standard“ in cases of cubital tunnel syndrome without anatomical pathologies that require further manipulation.

## Conclusion

The single ulnar nerve decompression is a safe and effective method to treat the cubital tunnel syndrome. It achieves significantly a reduction of pain and paresthesia, an increase of power and the motoric and sensitive function.

## Notes

### Competing interests

The authors declare that they have no competing interests.
